# SARS-CoV-2 Infection Is Asymptomatic in Nearly Half of Adults with Robust Anti-Spike Protein Receptor-Binding Domain Antibody Response

**DOI:** 10.3390/vaccines9030207

**Published:** 2021-03-02

**Authors:** Ourania E. Tsitsilonis, Dimitrios Paraskevis, Evi Lianidou, Evangelos Terpos, Athanasios Akalestos, Vassilios Pierros, Evangelia Georgia Kostaki, Efstathios Kastritis, Paraskevi Moutsatsou, Marianna Politou, Andreas Scorilas, Thomas Sphicopoulos, Nikolaos Thomaidis, Ioannis P. Trougakos, Athanassios Tsakris, Nikolaos Voulgaris, Christina C. Daskalaki, Zoi Evangelakou, Christina Fouki, Despoina D. Gianniou, Sentiljana Gumeni, Ioannis V. Kostopoulos, Maria S. Manola, Nikolaos Orologas-Stavrou, Chrysanthi Panteli, Eleni-Dimitra Papanagnou, Pantelis Rousakis, Aimilia D. Sklirou, Stavroula Smilkou, Dimitra Stergiopoulou, Sotirios Tsiodras, Meletios-Athanasios Dimopoulos, Petros P. Sfikakis

**Affiliations:** 1 Department of Biology, National and Kapodistrian University of Athens (NKUA), 15784 Athens, Greece; ascorilas@biol.uoa.gr (A.S.); itrougakos@biol.uoa.gr (I.P.T.); xristin1.dask@gmail.com (C.C.D.); zoievag@biol.uoa.gr (Z.E.); gndespoina@biol.uoa.gr (D.D.G.); sgumeni@biol.uoa.gr (S.G.); gikosto@gmail.com (I.V.K.); mmanola@biol.uoa.gr (M.S.M.); norologas@biol.uoa.gr (N.O.-S.); chrysanthipanteli23@gmail.com (C.P.); epapanagnou@biol.uoa.gr (E.-D.P.); rousakisp@gmail.com (P.R.); asklirou@biol.uoa.gr (A.D.S.); 2 Department of Hygiene, Epidemiology and Medical Statistics, School of Medicine, NKUA, 11527 Athens, Greece; ekostakh@med.uoa.gr (E.G.K.); chrfouki@hotmail.com (C.F.); 3 Department of Chemistry, NKUA, 15771 Athens, Greece; lianidou@chem.uoa.gr (E.L.); ntho@chem.uoa.gr (N.T.); ssmilkou@chem.uoa.gr (S.S.); dimitrastergiopoulou@yahoo.com (D.S.); 4 Department of Clinical Therapeutics, School of Medicine, Alexandra General Hospital, NKUA, 11528 Athens, Greece; eterpos@med.uoa.gr (E.T.); ekastritis@med.uoa.gr (E.K.); mdimop@med.uoa.gr (M.-A.D.); 5 Roche Diagnostics (Hellas) S.A., Marousi, 15125 Athens, Greece; thanasis.akalestos@roche.com; 6 Department of Informatics and Telecommunications, NKUA, 15784 Athens, Greece; pierrosv@di.uoa.gr (V.P.); thomas@di.uoa.gr (T.S.); 7 Department of Clinical Biochemistry, School of Medicine, University General Hospital Attikon, NKUA, 12462 Haidari, Greece; pmoutsatsou@med.uoa.gr; 8 Hematology Laboratory-Blood Bank, Aretaieio Hospital, School of Medicine, NKUA, 11528 Athens, Greece; mpolitou@med.uoa.gr; 9 Department of Microbiology, School of Medicine, NKUA, 11527 Athens, Greece; atsakris@med.uoa.gr; 10 Department of Geology and Geoenvironment, NKUA, 15784 Athens, Greece; voulgaris@geol.uoa.gr; 11 Fourth Department of Internal Medicine, School of Medicine, University Hospital Attikon, NKUA, 12462 Haidari, Greece; sotirios.tsiodras@gmail.com; 12 First Department of Propaedeutic Internal Medicine, School of Medicine, Laiko General Hospital, NKUA, 15772 Athens, Greece; psfikakis@med.uoa.gr

**Keywords:** SARS-CoV-2, seroepidemiology, asymptomatic, unsuspected/asymptomatic, antibodies

## Abstract

Between June and November 2020, we assessed plasma antibodies against severe acute respiratory syndrome coronavirus 2 (SARS-CoV-2) nucleocapsid protein in 4996 participants (aged 18–82 years, 34.5% men) from the National and Kapodistrian University of Athens. The weighted overall prevalence was 1.6% and monthly prevalence correlated with viral RNA-confirmed SARS-CoV-2 infections in Greece, in the same period. Notably, 49% of seropositive cases reported no history of SARS-CoV-2 infection-related clinical symptoms and 33% were unsuspected of their previous infection. Additionally, levels of anti-SARS-CoV-2 antibodies against the spike-protein receptor-binding domain were similar between symptomatic and asymptomatic individuals, irrespective of age and gender. Using Food and Drug Administration Emergency Use Authorization-approved assays, these results support the need for such studies on pandemic evaluation and highlight the development of robust humoral immune responses even among asymptomatic individuals. The high percentage of unsuspected/asymptomatic active cases, which may contribute to community transmission for more days than that of cases who are aware and self-isolate, underscores the necessity of measures across the population for the efficient control of the pandemic.

## 1. Introduction

One year after the description of the first clinical cases infected with the severe acute respiratory syndrome coronavirus 2 (SARS-CoV-2) in Wuhan China, the global pandemic has affected 190 countries and territories, caused over 70 million confirmed coronavirus disease 2019 (COVID-19) cases and over 1.5 million deaths [1]. However, SARS-CoV-2 infection is very heterogeneous, ranging from asymptomatic transmission to cases with mild/nonspecific or moderate symptoms and to severe, often life-threatening COVID-19 [2]; thus, it may be difficult to track asymptomatically infected individuals, and therefore avoid virus spread.

Asymptomatic and pre-symptomatic transmission is one of the major challenges in controlling the SARS-CoV-2 pandemic. Among adults infected with SARS-CoV-2, the proportion of those who experienced no signs of the disease and had no known contacts with a diagnosed or suspected COVID-19 case, is difficult to determine. These people are “innocently responsible” for SARS-CoV-2 transmission, whereas their contribution to the viral spread likely correlates with their numbers in given settings, reportedly being of smaller magnitude as compared with symptomatic COVID-19 patients [3]. To date, surveillance data following viral RNA detection have not been able to determine the extent of asymptomatic infection, with estimates ranging from very low (6%) to very high (96%), depending on the population studied and the period assessed [4]. According to a meta-analysis that included 79 studies (through to 10 June 2020) of 6616 people with PCR-diagnosed COVID-19 infection, about 20% remained asymptomatic during follow-up [3]. A second meta-analysis, through to 20 July 2020, of 21,708 at-risk people found that the proportion of asymptomatic cases was 17% overall, being higher in aged care (20%) and lower in non-aged care (16%) donors [5]. According to a study performed five to eight weeks after the end of lockdown in Wuhan, screening of the 9,865,404 participants without a history of COVID-19 did not find any newly confirmed COVID-19 cases and identified only 300 asymptomatic positive cases [6]. In another study describing clinical and demographic data in 4779 crew members, young men predominantly, of an aircraft carrier during an outbreak of COVID-19, PCR-positive asymptomatic infections were as high as 43% [7]. 

There is even fewer studies on asymptomatic infection based on anti-SARS-CoV-2 antibody measurements. Among 1847 participants working in three sites at an institution in Paris conurbation, of those detected immunoglobulin G (IgG)-positive for SARS-CoV-2 nucleocapsid (N)- and spike (S)-proteins, 21% had been asymptomatic [8]. Further evidence suggests that there is a positive correlation between the severity of clinical symptoms and serum levels of anti-SARS-CoV-2 antibodies, being less prominent in oligosymptomatic patients [9,10]. According to other reports, asymptomatic infection may not even induce a detectable humoral response, suggesting that high viral load is possibly associated with the levels of humoral immunity. Moreover, as previously described, antibody levels may decrease over time in patients with mild or moderate disease severity [11,12], and thus we may reasonably assume that, in asymptomatic individuals, a time-related decline of B cell responsiveness could lead to “serosilent” infections.

The aim of our study was to investigate the prevalence of antibodies against SARS-CoV-2 N-protein in active members from the National and Kapodistrian University of Athens and, among those who tested positive, to measure the levels of anti-S-protein receptor-binding domain (RBD) antibodies using a quantitative test. Moreover, we aimed at determining the characteristics and the symptomatic status of seropositive individuals and compared the antibody levels between the asymptomatic and symptomatic cases.

## 2. Materials and Methods

### 2.1. Blood Collection and Anti-SARS-CoV-2 Antibody Testing 

Blood sampling was conducted at selected locations within the National and Kapodistrian University of Athens (NKUA) facilities in Athens, Greece from June to November 2020. Samples were collected from 4996 randomly selected NKUA members, comprised of faculty members/laboratory assistants, scientific affiliates, administrative officers, undergraduate, and postgraduate students. Their demographic characteristics are shown in Table 1. The protocol was approved by the Ethics and Bioethics Committee of the School of Medicine, NKUA (protocol #312/02-06-2020) and study participants provided written informed consent.

In more detail, from June to November, members of the NKUA were offered plasma testing for SARS-CoV-2 antibodies. Their participation was voluntary, and donors were not selected based on symptoms or previous exposure to SARS-CoV-2. Prior to blood sampling, donors completed a brief epidemiological questionnaire and granted permission to access their clinical records. The questionnaire included professional information (e.g., status and position at the NKUA, location and type of work), demographic data (e.g., age, sex, and place of birth), medical history (e.g., previous vaccination, administration of drugs, and chronic medical conditions such as diabetes, cardiomyopathy, and chronic obstructive pulmonary disease), and direct questions on whether they had been diagnosed (e.g., via PCR testing and/or hospitalization) or presented any of the most characteristic COVID-19 symptoms (e.g., fever of any grade, fatigue, conjunctivitis, sweating, cough, headache, respiratory distress/dyspnea, smell or taste loss, and diarrhea) or had contact with known or suspected COVID-19 individuals, and the interval from contact to blood donation. All donors were asked to voluntarily provide a second sample after 3 months, of whom 988 agreed. Cases were considered to be asymptomatic in the absence of any COVID-19-related symptoms during the previous months, as reported on the self-assessed questionnaire, which was additionally confirmed via a personal interview by a physician following the positive anti-SARS-CoV-2 result.

Peripheral blood was collected, treated, and stored, as previously described [13]. Briefly, after venipuncture, blood was transferred to K2EDTA vacutainers (BD Biosciences, San Jose, CA, USA) and centrifuged at 500× g for 20 min within 3 h from collection. Plasma was transferred into DNA-RNA free cryovials (Corning, NY, USA), immediately frozen at −20 °C, and analyzed within 20 days post storage. All samples (4996 initially tested and 988 retested) were analyzed using the CE-IVD Roche Elecsys^®^ Anti-SARS-CoV-2 test, an electrochemiluminescence immunoassay (ECLIA) for the detection of total antibodies (IgG, IgM, and IgA (pan-Ig)) to SARS-CoV-2 N-protein (Roche Diagnostics GmbH, Mannheim, Germany) [14]. The assay uses, as antigen, the recombinant N-protein of SARS-CoV-2 and the method is based on a “double-antigen sandwich” format between donors’ antibodies, biotinylated SARS-CoV-2 N-antigen, and SARS-CoV-2 recombinant N-antigen labeled with a ruthenium complex. After concomitant incubation of the reagents, streptavidin-coated microparticles are added; the immune-complex binds to the solid phase via a biotin-streptavidin interaction, aspirated into the measuring cell of the Roche Cobas e411 analyzer, and microparticles are magnetically captured onto the electrode surface. Unbound material is washed out and the emission of chemiluminescence is measured by a photomultiplier. The total procedure required 20 μL of plasma and the duration of the assay was 18 min. Test results were generated by interpolating the ECLIA signal with that of a threshold generated during calibration. The results were expressed as qualitative statements (reactive/non-reactive), with a cutoff index (COI) ≥1.0 considered to be reactive. According to the manufacturer’s package insert, Elecsys^®^ Anti-SARS-CoV-2 exhibits overall clinical specificity of 99.81% with no cross-reactivity to the common cold coronaviruses and clinical sensitivity of 100% for samples collected ≥14 days after PCR confirmation [14]. Positive plasma samples with a COI ≥ 1.0, as well as selected samples with COI ≥ 0.1, or samples derived from volunteers reporting COVID-19-like symptoms were subsequently analyzed with the CE-IVD Roche Elecsys^®^ Anti-SARS-CoV-2 S, an ECLIA for the quantitative determination of antibodies (including IgGs) to the SARS-CoV-2 S-protein RBD (Roche Diagnostics) [15]. This assay uses a recombinant protein representing the RBD of the S antigen and the method is the same as aforementioned for the Anti-SARS-CoV-2 ECLIA, except for mixing donors’ samples with biotinylated and ruthenylated RBD antigen and the analyzer used in this assay was the Roche Cobas e801. The results were expressed as quantitative statements (in Units (U)/mL), and samples with ≥0.8 U/mL were considered to be positive. According to the manufacturer’s package insert, Elecsys^®^ Anti-SARS-CoV-2 S exhibits overall clinical specificity of 100% and clinical sensitivity of 98.8% for samples collected ≥14 days after PCR confirmation [15]. Both tests have been Food and Drug Administration Emergency Use Authorization (FDA-EUA) approved and were performed according to the manufacturer’s instructions.

### 2.2. Statistical Analysis

The statistical analysis was performed on STATA 13-StataCorp LP and GraphPad Prism version 8.0.2 (GraphPad software, La Jolla, CA, USA). Data were summarized using median and interquartile ranges (continuous variables), and absolute and relative frequencies (categorical variables). Simple comparisons of the relevant distributions across different levels of other categorical variables were based on non-parametric tests (Mann-Whitney U Test). Putative correlations were assessed by Spearman’s correlation coefficient. All analyses were two-sided and statistical significance was assumed at *p* < 0.05.

The prevalence of antibodies against SARS-CoV-2 was initially computed by calculating the unweighted proportions of positive tests (unweighted prevalence). Then, the final estimation of the prevalence was obtained by the following two-step approach: First, the unweighted proportions of positive tests were adjusted for the sensitivity and specificity of the test, according to the manufacturer’s specification, as implemented in the epiR package (R version 3.6.3, R Foundation for Statistical Computing, Vienna, Austria); secondly, the prevalence was estimated, after weighting adjustments for the age distribution (18–82 years old) of the population in the Attica region (data from the 2011 census).

The incidence seroconversion rate was estimated using data from the NKUA participants who provided 2 samples at a 3-month interval and who tested negative for antibodies against the N-protein of SARS-CoV-2 at their first sample. The seroconversion time was estimated by the midpoint of the interval between the last negative and the first positive sample.

## 3. Results

### 3.1. Seroepidemiology 

#### 3.1.1. Study Population

A total of 4996 volunteers from the NKUA participated in the study. Seventy of the 4996 volunteers were found to be positive for antibodies against the N-protein of SARS-CoV-2. Furthermore, nine additional seroconversions were found among the 988 individuals who were retested after approximately 3 months (time at risk 264 person-years), yielding an overall seroconversion incidence rate of 3.40 new cases per 100 person-years (95% CI 1.77, 6.54). Although PCR testing of participants was not performed in the frame of the current survey, it was of interest that of the 79 antibody-positive cases, 34 (43%) cases had a PCR test performed during the past months on a private basis. Of these cases, confirmed SARS-CoV-2 infection was reported in only six (17.6%) cases, as stated in the self-assessed questionnaire and confirmed during their personal interview with a physician. 

#### 3.1.2. Seroprevalence Study

**Figure 1:**
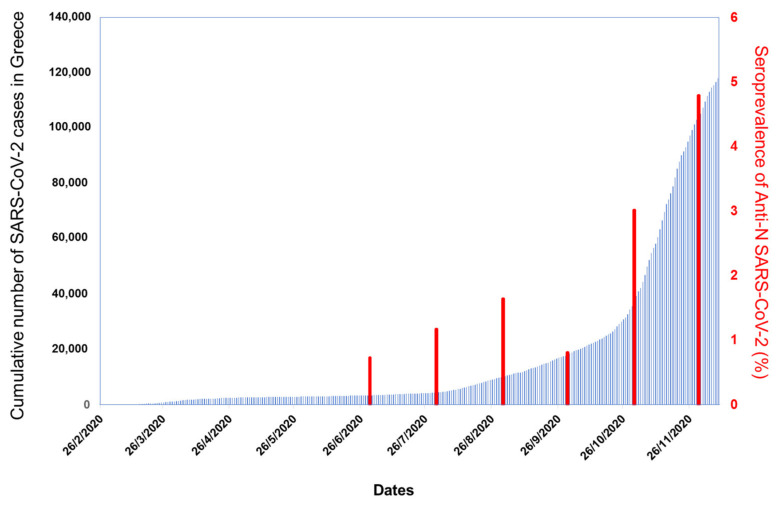
Seroprevalence of SARS-CoV-2 antibodies for each month of the survey and cumulative case counts per day in Greece. Blue bars show the cumulative number of SARS-CoV-2 cases recorded in Greece per day from 26 February to 4 December 2020. Red bars show the weighted seroprevalence for age and test performance estimated for the anti-SARS-CoV-2 N-protein per month (July–November) in the volunteer participants from the National and Kapodistrian University of Athens (NKUA).

Overall, the unweighted seroprevalence of anti-SARS-CoV-2 N-protein antibodies was 1.58% and, after adjusting for age and the indicated sensitivity (100%) and specificity (99.81%) of the test’s package insert, the weighted seroprevalence was 1.60% (95% CI 0.92, 2.54; Table 2). The weighted seroprevalence for different group categories according to gender, age, place of employment, and status at the NKUA is also shown in Table 2 and Figure S1. No significant difference was observed between the weighted seroprevalence in men (2.00% (95% CI 0.87, 3.79)) and women (1.31% (95% CI 0.57, 2.51), *p* = 0.061). Differences were observed among the NKUA participants, with students having significantly lower weighted seroprevalence (0.77% (95% CI 0.34, 5.44)) than scientific affiliates (2.48% (95% CI 0.71, 6.20), *p* = 0.0001); no significant differences were found between students and faculty members/laboratory assistants (1.15% (95% CI 0.29, 23.50), *p* = 0.376) or administrative officers (1.13% (95% CI 0.31, 4.40), *p* = 0.315). Although the weighted seroprevalence in those belonging to the age group of 55–82 years was higher (2.46% (95% CI 1.33, 4.09)), no significant differences were observed between them and those belonging to the age group of 18–34 years (1.34% (95% CI 0.91, 1.89), *p* = 0.766) or 35–54 years (1.05% (95% CI 0.57, 1.70), *p* = 0.739). The weighted seroprevalence for the School of Health Sciences (1.87% (95% CI 0.66, 3.99)) was higher than for non-health sciences departments (1.46% (95% CI 0.56, 3.03)) and other facilities (1.51% (95% CI 0.31, 11.27)); however, these differences were not statistically significant. Furthermore, a non-significant increasing trend was observed for the weighted seroprevalence with time in the six consecutive months of the survey (*p* = 0.064, Supplementary Figure S1), following the overall course of the pandemic in the country. As shown in Figure 1, the monthly seroprevalence of antibodies against the N-protein of SARS-CoV-2 in the NKUA cohort of 4996 participants followed the national trend of the SARS-CoV-2 confirmed cases between June and November 2020.

### 3.2. Characteristics of Anti-SARS-CoV-2 Positive Individuals

Interestingly, SARS-CoV-2 infection had been symptomatic in only 40/79 antibody-positive active participants, as reported on the self-assessed questionnaire and confirmed by the physician’s personal interview; 2/40 stated previous admission in the hospital, whereas oxygen supplementation was required for one of them. In the remaining NKUA participants positive for anti-SARS-CoV-2 N-protein antibodies (39/79, 49.4%), a presumable asymptomatic infection was reported (Table 3). As aforementioned, infection was considered to be asymptomatic in the absence of any COVID-19-related symptoms (fever of any grade, fatigue, conjunctivitis, sweating, headache, cough, dyspnea, diarrhea, taste loss, and smell loss) reported during the previous months. Although lack of symptoms was initially evaluated according to the self-reported questionnaire, all asymptomatic cases with positive anti-SARS-CoV-2 results were further individually confirmed by a physician’s personal interview. Moreover, the majority of SARS-CoV-2 asymptomatic infections were “unsuspected” cases (26/39, 66.7%), i.e., there was no known contact reported with any confirmed or suspected COVID-19 case. The remaining 40/79 cases that tested positive for anti-SARS-CoV-2 antibodies were symptomatic (50.6%), i.e., reported at least one COVID-19-related symptom.

Importantly, no difference was found in the asymptomatic infection rate with age. Specifically, the proportion of asymptomatic infection was 40.5% (15 out of 37) for the age group 18–34 years, 61.1% (11 out of 18) for the age group 35–54 years, and 40.0% (6 out of 15) for the age group 55–82 years. No significant differences were observed between the age groups (18–34 vs. 35–54 years, *p* = 0.2993; 18–34 vs. 55–82 years, *p* = 0.9832; 35–54 vs. 55–82 years, *p* = 0.4044). 

### 3.3. Quantitative Detection of Anti-SARS-CoV-2 Antibodies

**Figure 2:**
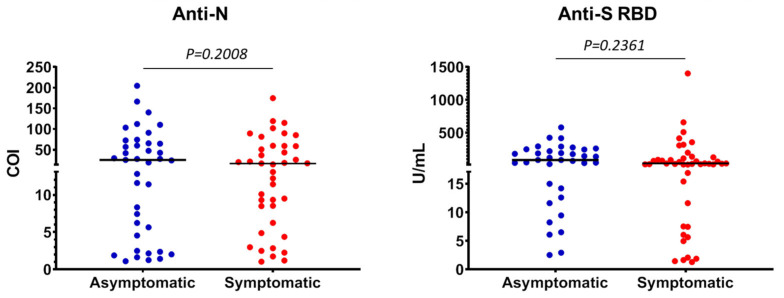
Plasma levels of anti-N (left) and anti-S RBD (right) antibodies in participants from the National and Kapodistrian University of Athens (NKUA) with asymptomatic and symptomatic SARS-CoV-2 infection. Each circle represents values (cutoff index (COI) or Units (U)/mL) from a single participant. Horizontal bars indicate median values (Mann-Whitney test).

**Figure 3:**
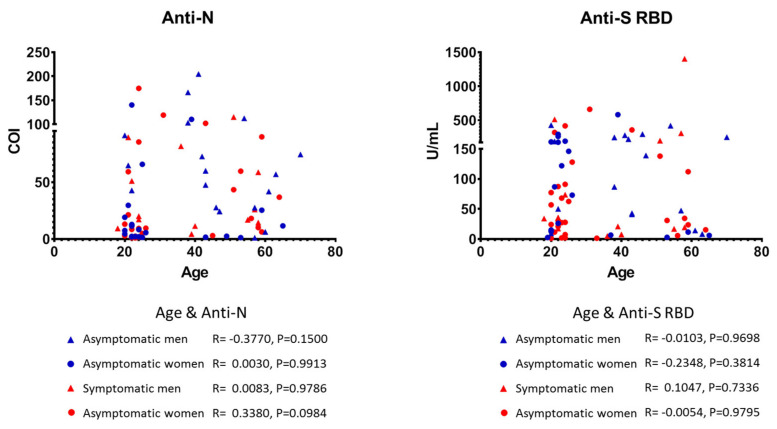
Scatter plots of SARS-CoV-2 anti-N (left) and anti-S RBD (right) antibody plasma levels of the participants from the National and Kapodistrian University of Athens (NKUA) with asymptomatic (blue symbols) and symptomatic (red symbols) SARS-CoV-2 infection stratified by gender (triangles, men; circles, women). Each symbol represents values (COI or U/mL) from a single participant (Spearman test).

To examine plasma concentrations of anti-SARS-CoV-2 antibodies in our study population, we reanalyzed samples that tested positive in the anti-N assay, as well as samples with COI values between 0.1 and 1, by a quantitative newly approved assay for antibodies against the RBD of the SARS-CoV-2 S-protein. Using the latter test, 79 samples were found positive for anti-SARS-CoV-2 S-protein antibodies. The level of concordance between the two different assays (anti-N vs. anti-S RBD) was 93.4% (253/271, with 70 samples found positive by both assays). Again, among nearly half of the donors, namely 35/79 (44.3%), prior infection was asymptomatic (Table 3) and the majority of these donors were unsuspected of their SARS-CoV-2 infection. Among the 70 plasma samples tested positive with both methods, 32 (45.7%) and 38 (54.3%) belonged to asymptomatic and symptomatic individuals, respectively (Table 3). Interestingly, nine of 18 samples (50%) with the highest anti-S RBD antibody levels, i.e., those exceeding the upper detection limit of the test (250 U/mL) belong to the asymptomatic cases. Overall, antibody levels against S-protein RBD were similar between asymptomatic and symptomatic cases (Figure 2), suggesting that the intensity of humoral responses against SARS-CoV-2 antigens is independent of notable clinical expression of the infection. In support, and under the limitation that the anti-N assay used herein is not quantitative, the COI levels of asymptomatic individuals were comparable to the COIs of donors who developed clinical symptoms (Figure 2). Finally, no significant correlations were found between the levels of either anti-N or anti-S RBD antibodies with age, gender or the symptomatic status of infection in our study (Figure 3). 

## 4. Discussion

During the first SARS-CoV-2 pandemic wave, Greece was among the less affected countries. However, in our cohort comprising 4996 participants from the NKUA the weighted seroprevalence increased from June to November (Figure 1), a finding that is in accordance with the surge in SARS-CoV-2 cases observed after the middle of October in Greece. Specifically, the highest percentage of anti-SARS-CoV-2 prevalence was detected in November, in the midst of the second wave of the pandemic in Greece, when the highest number of SARS-CoV-2 PCR-positive diagnoses was reported. Retesting of 988 participants, after 3 months, i.e., during October and November 2020, revealed an overall seroconversion incidence rate of 3.40 new cases per 100 person-years, which was higher than the seroprevalence estimated for the previous months. In our interim analysis, the weighted seroprevalence for volunteers tested until July 2020 was 0.93% [13]. 

Herein, we also found that nearly half of the seropositive individuals, as assessed by two different anti-SARS-CoV-2 assays, were asymptomatic. Notably, the majority of them were also unsuspected (i.e., reported no contact with confirmed or suspected COVID-19 cases). This finding is both clinically and epidemiologically relevant, as Greece, and accordingly our NKUA cohort, were less affected by the pandemic. Previous seroprevalence studies in populations other than healthy blood donors [16] or health care workers [17] have reported that asymptomatic cases did not exceed a mean percentage of 20%, although this number varied greatly among different cohorts [17]. For example, in a study which analyzed 7770 close contacts (1863 household, 2319 work, and 3588 social contacts) of 1114 PCR-confirmed index cases in Singapore, 36% of individuals with SARS-CoV-2 infection were reported as asymptomatic [18]. Similarly, in a nationwide study in Spain including more than 60,000 participants, the proportion of seropositive who were asymptomatic ranged between 21.9% and 35.8% [19]. The percentage of asymptomatic cases was higher in cohorts that tested PCR-positive in heavily affected areas during the first wave, for example, in Vo’, Italy (42.5%) [20], and more pronounced in outbreaks in “isolated” populations, such as among the Diamond Princess ship crew (more than 50%) [21], or in detained persons (63%) [22], where superspreading events can take place due to particular conditions.

We also found that the levels of anti-SARS-CoV-2 antibodies were not different between asymptomatic and symptomatic cases (Figure 2). Moreover, no differences were found in antibody levels stratified by gender or age or in the rates of asymptomatic infection with age. To the best of our knowledge, this is one of the few studies showing that the levels of the adaptive humoral immune response to SARS-CoV-2 expressed as plasma concentrations of antibodies do not correlate with the presence of symptomatic disease. In contrast, others have suggested that factors such as high viral load, disease severity, and older age likely correlate with increased responsiveness of humoral immunity; however, these studies included hospitalized COVID-19 patients [11,23,24,25]. Interestingly, in their most recent study, Sasisekharan et al. [26] showed that anti-S1 and anti-RBD antibody levels could differentiate mild from moderate and severe cases, whereas anti-N antibodies could not clearly distinguish these categories, suggesting that associations between antibody titers and COVID-19 severity likely depend on the SARS-CoV-2-specific immunoassay used. Furthermore, Grossberg et al. [27] showed that IgG responses were significantly greater in symptomatic than asymptomatic participants, whereas asymptomatic cases had higher IgM titers. This newly published observation supports our results, since both assays used in our study for determining anti-N and anti-RBD specific antibodies detected all Ig classes (pan-Ig ECLIAs), and thus the similar antibody levels of asymptomatic and symptomatic cases might likely reflect a different Ig class predominance. 

Overall, our results indicate that asymptomatic and mostly unsuspected SARS-CoV-2 infections are more prevalent than currently thought. Moreover, the number of asymptomatic cases in the community could be even higher, since the seroprevalence in cross-sectional studies can be underestimated without adjustment for waning antibodies, as previously reported in studies from New York City and Connecticut [28]. 

On the one hand, our study has some limitations, the most important being that asymptomatic cases were self-reported. Although the self-assessed questionnaire was very detailed and the lack of COVID-19-related symptoms were individually confirmed by a physician’s interview, mild symptoms in some cases may have been overlooked. Additionally, the study population does not provide a representative sample of the population living in Attica or Greece, since it was not based on random sampling. However, the monthly seroprevalence followed the trend in the number of infections across the country. Moreover, herein, identification of SARS-CoV-2 infection was based solely on antibody testing and not on PCR results, since molecular testing of the participants was not performed within the frame of this study; accordingly, among antibody-positive individuals only one out of seven reported a PCR-confirmed SARS-CoV-2 infection, assessed on a private basis. Nonetheless, molecular testing can identify only current and not recovered (past) infections. Massive antigen and molecular screening can provide a momentary view of the infection rates but may have less sensitivity and does not provide estimates of humoral immunity at the population level. Moreover, it is impossible to estimate the exact time of infection in asymptomatic individuals, which accordingly influences the result of antibody measurements, as their levels seem to reduce with time.

On the other hand, although there is no optimal strategy to precisely identify asymptomatic SARS-CoV-2 infected individuals, our study complies with most of the recommended methods proposed by Meyerowitz et al. [29]. Firstly, our survey was performed among trustful University members who may recall COVID-19-related symptoms reliably. Secondly, questionnaires included a broad symptom definition, and a physician was constantly present during blood sampling to provide necessary explanations to every participant, whereas the medical history of positive cases was subsequently confirmed by a physician’s telephone interview. Thirdly, the serological assays performed are among those with the highest performance characteristics, both FDA-EUA approved, and the second quantitative assay confirmed positive samples. Finally, our study was designed and executed by faculty members of the NKUA School of Medicine, highly experienced in clinical trials/studies. 

## 5. Conclusions

Beyond the weighted overall seroprevalence rates and the dynamic monthly seroprevalence that followed the trend of the confirmed SARS-CoV-2 cases in Greece during the same period, in this study, we show that a high proportion of SARS-CoV-2 infected individuals are asymptomatic and display a robust adaptive humoral response, implying a possibly earlier than predicted development of herd immunity in various communities. By extrapolating our results, the true number of people who developed adaptive humoral immune responses against SARS-CoV-2 may be at least two times higher than the estimates based on symptomatic cases. More importantly, the high rates of unsuspected SARS-CoV-2 infections among socially and professionally active participants suggest that these people act as “cryptic sources” for viral spreading and may contribute to community transmission for more days than people who are aware of a probable infection and become self-isolated. This should inform nationwide healthcare policies, highlighting the necessity of public health measures, such as social distancing and use of protective masks for the entire population to control the pandemic.

## Figures and Tables

**Table 1:** Study population and sociodemographic characteristics.

Characteristic	Number of Participants	Percentage (%)
**Overall**	4996	100.0
**Gender**		
Male	1726	34.5
Female	3270	65.4
**Age group (in years)**		
(18–34)	2659	53.2
(35–54)	1688	33.8
(55–82)	602	12.1
Not reported	47	0.9
**Status at the NKUA**		
Undergraduate/postgraduate students	2722	54.5
Scientific affiliates	751	15.0
Faculty members/laboratory assistants	536	10.7
Administrative officers	874	17.5
Not reported	113	2.3

**Table 2:** Weighted prevalence for age and test performance of anti-severe acute respiratory syndrome coronavirus 2 (anti-SARS-CoV-2) nucleocapsid (N)-protein antibodies.

Characteristic	Number of Anti-SARS-CoV-2 Positive Cases	Unweighted Prevalence (%)	Weighted Prevalence (%) (95% Confidence Interval)
**Overall**	79	1.58	1.60 (0.92, 2.54) *
**Gender**			
Male	31	1.80	2.00 (0.87, 3.79)
Female	48	1.48	1.31 (0.57, 2.51)
Unknown	0	-	-
**Age group (in years)**			
(18–34)	41	1.54	1.34 (0.91, 1.89)
(35–54)	21	1.24	1.05 (0.57, 1.70)
(55–82)	16	2.66	2.46 (1.33, 4.09)
Unknown	1	-	-
**School of NKUA**			
Health Sciences	26	1.57	1.87 (0.66, 3.99)
Non-health sciences	40	1.72	1.46 (0.56, 3.03)
Other facility	7	2.43	1.51 (0.31, 11.27)
Unknown	6	-	-
**Status at the NKUA**			
Undergraduate/postgraduate Students	44	1.62	0.77 (0.34, 5.44)
Scientific affiliates	11	1.50	2.48 (0.71, 6.20)
Faculty members/ laboratory assistants	11	2.05	1.15 (0.29, 23.50)
Administrative officers	13	1.52	1.13 (0.31, 4.40)
Unknown	0	-	-

* Missing values for age, 47 out of 4996 participants (N = 4949).

**Table 3:** Symptomatic status of participants positive for SARS-CoV-2 antibodies against N-protein, and receptor-binding domain (RBD).

SARS-CoV-2 Antibodies		Asymptomatic			Symptomatic		
	Total *n*	*n* (%)	Median Age in Years (Range)	Male, *n* (%)	*n* (%)	Median Age in Years (Range)	Male *n* (%)
Anti-N positive	79	39 (49.4)	38 (20–70)	18 (46.2)	40 (50.6)	24.5 (18–64)	13 (32.5)
Anti-S RBD positive	79	35 (44.3)	37 (19–70)	17 (48.6)	44 (55.7)	24 (18–64)	14 (31.8)
Anti-N & anti-S RBD positive	70	32 (45.7)	38 (20–70)	16 (50.0)	38 (54.3)	24.5 (18–64)	13 (34.2)

## Data Availability

The data presented in this study are available on request from the corresponding author. The data are not publicly available due to restrictions on privacy.

## Supplementary Materials

The following are available online at https://www.mdpi.com/2076-393X/9/3/207/s1, Figure S1: Weighted seroprevalence for age and test performance of anti-SARS CoV-2 N-protein per gender, age group (in years), School of NKUA (HS: Health Sciences, Non-HS: Non-health sciences), position at the NKUA and per month (June-November 2020).

## References

[1] World Health Organization (2020). Weekly Epidemiological Update—15 December 2020.

[2] Fajgenbaum D.C., June C.H. (2020). Cytokine storm. N. Engl. J. Med..

[3] Bi Q., Lessler J., Eckerle I., Lauer S.A., Kaiser L., Vuilleumier N., Cummings D.A.T., Flahault A., Petrovic D., Guessous I. (2020). Household transmission of SARS-CoV-2: Insights from a population-based serological survey. MedRxiv Preprint.

[4] Buitrago-Garcia D., Egli-Gany D., Counotte M.J., Hossmann S., Imeri H., Ipekci A.M., Salanti G., Low N. (2020). Occurrence and transmission potential of asymptomatic and presymptomatic SARS-CoV-2 infections: A living systematic review and meta-analysis. PLoS Med..

[5] Byambasuren O., Cardona M., Bell K., Clark J., McLaws M.-L., Glasziou P. (2020). Estimating the extent of asymptomatic COVID-19 and its potential for community transmission: Systematic review and meta-analysis. JAMMI.

[6] Cao S., Gan Y., Wang C., Bachmann M., Wei S., Gong J., Huang Y., Wang T., Li L., Lu K. (2020). Post-lockdown SARS-CoV-2 nucleic acid screening in nearly ten million residents of Wuhan, China. Nat. Commun..

[7] Kasper M.R., Geibe J.R., Sears C.L., Riegodedios A.J., Luse T., Von Thun A.M., McGinnis M.B., Olson N., Houskamp D., Fenequito R. (2020). An outbreak of Covid-19 on an aircraft carrier. N. Engl. J. Med..

[8] Anna F., Goyard S., Lalanne A.I., Nevo F., Gransagne M., Souque P., Louis D., Gillon V., Turbiez I., Bidard F.-C. (2021). High seroprevalence but short-lived immune response to SARS-CoV-2 infection in Paris. Eur. J. Immunol..

[9] Graham N.R., Whitaker A.N., Strother C.A., Miles A.K., Grier D., McElvany B.D., Bruce E.A., Poynter M.E., Pierce K.K., Kirkpatrick B.D. (2020). Kinetics and isotype assessment of antibodies targeting the spike protein receptor-binding domain of severe acute respiratory syndrome-coronavirus-2 in COVID-19 patients as a function of age, biological sex and disease severity. Clin. Transl. Immunol..

[10] Ripperger T.J., Uhrlaub J.L., Watanabe M., Wong R., Castaneda Y., Pizzato H.A., Thompson M.R., Bradshaw C., Weinkauf C.C., Bime C. (2020). Orthogonal SARS-CoV-2 serological assays enable surveillance of low-prevalence communities and reveal durable humoral immunity. Immunity.

[11] Chen Y., Zuiani A., Fischinger S., Mullur J., Atyeo C., Travers M., Lelis F.J.N., Pullen K.M., Martin H., Tong P. (2020). Quick COVID-19 healers sustain anti-SARS-CoV-2 antibody production. Cell.

[12] Terpos E., Mentis A., Dimopoulos M.A. (2020). Loss of anti-SARS-CoV-2 antibodies in mild Covid-19. N. Engl. J. Med..

[13] Tsitsilonis O.E., Paraskevis D., Lianidou E., Pierros V., Akalestos A., Kastritis E., Moutsatsou P., Scorilas A., Sphicopoulos T., Terpos E. (2020). Seroprevalence of antibodies against SARS-CoV-2 among the personnel and students of the National and Kapodistrian University of Athens, Greece: A preliminary report. Life.

[14] Elecsys Anti-SARS-CoV-2 Insert, Roche Diagnostics GmbH, Sandhofer Strasse 116, D-68305 Mannheim. www.roche.com.

[15] Elecsys Anti-SARS-CoV-2 S Insert, Roche Diagnostics GmbH, Sandhofer Strasse 116, D-68305 Mannheim. www.roche.com.

[16] Slot E., Hogema B.M., Reusken C.B.E.M., Reimerink J., Molier M., Karregat J.H.M., IJlst J., Novotny V.M.J., VanLier R.A.W., Zaaijer H.L. (2020). Low SARS-CoV-2 seroprevalence in blood donors in the early COVID-19 epidemic in the Netherlands. Nat. Commun..

[17] Garcia-Basteiro A.L., Moncunill G., Tortajada M., Vidal M., Guinovart C., Jiménez A., Santano R., Sanz S., Méndez S., Llupià A. (2020). Seroprevalence of antibodies against SARS-CoV-2 among health care workers in a large Spanish reference hospital. Nat. Commun..

[18] Ng O.T., Marimuthu K., Koh V., Pang J., Linn K.Z., Sun J., De Wang L., Chia W.N., Tiu C., Chan M. (2020). SARS-CoV-2 seroprevalence and transmission risk factors among high-risk close contacts: A retrospective cohort study. Lancet Infect. Dis..

[19] Pollán M., Pérez-Gómez B., Pastor-Barriuso R., Oteo J., Hernán M.A., Pérez-Olmeda M., Sanmartín J.L., Fernández-García A., Cruz I., Fernández de Larrea N. (2020). Prevalence of SARS-CoV-2 in Spain (ENE-COVID): A nationwide, population-based seroepidemiological study. Lancet.

[20] Lavezzo E., Franchin E., Ciavarella C., Cuomo-Dannenburg G., Barzon L., Del Vecchio C., Rossi L., Manganelli R., Loregian A., Navarin N. (2020). Suppression of a SARS-CoV-2 outbreak in the Italian municipality of Vo’. Nature.

[21] Emery J.C., Russell T.W., Liu Y., Hellewell J., Pearson C.A., Knight G.M., Eggo R.M., Kucharski A.J., Funk S., CMMID COVID-19 Working Group (2020). The contribution of asymptomatic SARS-CoV-2 infections to transmission on the Diamond Princess cruise ship. Elife.

[22] Wadhwa A., Fisher K.A., Silver R., Koh M., Arons M.M., Miller D.A., McIntyre A.F., Vuong J.T., Kim K., Takamiya M. (2021). Identification of presymptomatic and asymptomatic cases using cohort-based testing approaches at a large correctional facility—Chicago, Illinois, USA, May 2020. Clin. Infect. Dis..

[23] Hashem A.M., Algaissi A., Almahboub S.A., Alfaleh M.A., Abujamel T.S., Alamri S.S., Alluhaybi K.A., Hobani H.I., AlHarbi R.H., Alsulaiman R.M. (2020). Early humoral response correlates with disease severity and outcomes in COVID-19 patients. Viruses.

[24] Li K., Huang B., Wu M., Zhong A., Li L., Cai Y., Wang Z., Wu L., Zhu M., Li J. (2020). Dynamic changes in anti-SARS-CoV-2 antibodies during SARS-CoV-2 infection and recovery from COVID-19. Nat. Commun..

[25] Terpos E., Politou M., Sergentanis T.N., Mentis A., Rosati M., Stellas D., Bear J., Hu X., Felber B.K., Pappa V. (2020). Anti-SARS-CoV-2 antibody responses in convalescent plasma donors are increased in hospitalized patients; subanalyses of a phase 2 clinical study. Microorganisms.

[26] Sasisekharan V., Pentakota N., Jayaraman A., Tharakaraman K., Wogan G.N., Narayanasami U. (2021). Orthogonal immunoassays for IgG antibodies to SARS-CoV-2 antigens reveal that immune response lasts beyond 4 mo post illness onset. Proc. Natl. Acad. Sci. USA.

[27] Grossberg A.N., Koza L.A., Ledreux A., Prusmack C., Krishnamurthy H.K., Jayaraman V., Granholm A.C., Linseman D.A. (2021). A multiplex chemiluminescent immunoassay for serological profiling of COVID-19-positive symptomatic and asymptomatic patients. Nat. Commun..

[28] Shioda K., Lau M.S., Kraay A.N., Nelson K.N., Siegler A.J., Sullivan P.S., Collins M.H., Weitz J.S., Lopman B.A. (2020). Estimating the cumulative incidence of SARS-CoV-2 infection and the infection fatality ratio in light of waning antibodies. medRxiv.

[29] Meyerowitz E.A., Richterman A., Bogoch I.I., Low N., Cevik M. (2020). Towards an accurate and systematic characterisationofpersistently asymptomatic infection with SARS-CoV-2. Lancet Infect. Dis..

