# Supplementary material for: SARS-CoV-2 Infection Is Asymptomatic in Nearly Half of Adults with Robust Anti-Spike Protein Receptor-Binding Domain Antibody Response
Source: Vaccines (Basel). 2021 Mar 2;9(3):207. doi: 10.3390/vaccines9030207 (PMC7998869; doi:10.3390/vaccines9030207)
Supplement: Supplementary file 1 [file vaccines-09-00207-s001.pdf]

vaccines

MDPI

Supplementary Materials

SARS-CoV-2 Infection Is Asymptomatic in Nearly Half of Adults with Robust Anti-Spike Protein Receptor-Binding Domain Antibody Response

Ourania E. Tsitsilonis [1,*,†], Dimitrios Paraskevis [2,*,†], Evi Lianidou [3], Evangelos Terpos [4], Athanasios Akalestos [5], Vassilios Pierros [6], Evangelia Georgia Kostaki [2], Efstathios Kastritis [4], Paraskevi Moutsatsou [7], Marianna Politou [8], Andreas Scorilas [1], Thomas Sphicopoulos [6], Nikolaos Thomaidis [3], Ioannis P. Trougakos [1], Athanassios Tsakris [9], Nikolaos Voulgaris [10], Christina C. Daskalaki [1], Zoi Evangelakou [1], Christina Fouki [2], Despoina D. Gianniou [1], Sentiljana Gumeni [1], Ioannis V. Kostopoulos [1], Maria S. Manola [1], Nikolaos Orologas-Stavrou [1], Chrysanthi Panteli [1], Eleni-Dimitra Papanagnou [1], Pantelis Rousakis [1], Aimilia D. Sklirou [1], Stavroula Smilkou [3], Dimitra Stergiopoulou [3], Sotirios Tsiodras [11], Meletios-Athanasios Dimopoulos [4] and Petros P. Sfikakis [12]

[1] Department of Biology, National and Kapodistrian University of Athens (NKUA), 15784 Athens, Greece; ascorilas@biol.uoa.gr (A.S.); itrougakos@biol.uoa.gr (I.P.T.); xristin1.dask@gmail.com (C.C.D.); zoievag@biol.uoa.gr (Z.E.); gndespoina@biol.uoa.gr (D.D.G.); sgumeni@biol.uoa.gr (S.G.); gikosto@gmail.com (I.V.K.); mmanola@biol.uoa.gr (M.S.M.); norologas@biol.uoa.gr (N.O.-S.); chrysanthipanteli23@gmail.com (C.P.); epapanagnou@biol.uoa.gr (E.-D.P.); rousakisp@gmail.com (P.R.); asklirou@biol.uoa.gr (A.D.S.)
[2] Department of Hygiene, Epidemiology and Medical Statistics, School of Medicine, NKUA, 11527 Athens, Greece; ekostakh@med.uoa.gr (E.G.K.); chrfouki@hotmail.com (C.F.)
[3] Department of Chemistry, NKUA, 15771 Athens, Greece; lianidou@chem.uoa.gr (E.L.); ntho@chem.uoa.gr (N.T.); ssmilkou@chem.uoa.gr (S.S.); dimitrastergiopoulou@yahoo.com (D.S.)
[4] Department of Clinical Therapeutics, School of Medicine, Alexandra General Hospital, NKUA, 11528 Athens, Greece; eterpos@med.uoa.gr (E.T.); ekastritis@med.uoa.gr (E.K.); mdimop@med.uoa.gr (M.-A.D.)
[5] Roche Diagnostics (Hellas) S.A., Marousi, 15125 Athens, Greece; thanasis.akalestos@roche.com
[6] Department of Informatics and Telecommunications, NKUA, 15784 Athens, Greece; pierrosv@di.uoa.gr (V.P.); thomas@di.uoa.gr (T.S.)
[7] Department of Clinical Biochemistry, School of Medicine, University General Hospital Attikon, NKUA, 12462 Haidari, Greece; pmoutsatsou@med.uoa.gr
[8] Hematology Laboratory-Blood Bank, Aretaieio Hospital, School of Medicine, NKUA, 11528 Athens, Greece; mpolitou@med.uoa.gr (M.P.)
[9] Department of Microbiology, School of Medicine, NKUA, 11527 Athens, Greece; atsakris@med.uoa.gr
[10] Department of Geology and Geoenvironment, NKUA, 15784 Athens, Greece; voulgaris@geol.uoa.gr
[11] Fourth Department of Internal Medicine, School of Medicine, University Hospital Attikon, NKUA, 12462 Haidari, Greece; sotirios.tsiodras@gmail.com
[12] First Department of Propaedeutic Internal Medicine, School of Medicine, Laiko General Hospital, NKUA, 15772 Athens, Greece; psfikakis@med.uoa.gr
* Correspondence: rtsitsil@biol.uoa.gr (O.E.T.); dparask@med.uoa.gr (D.P.); Tel: +30-2107274215 (O.E.T.); +30-2107462119 (D.P.)
† The authors contributed equally.

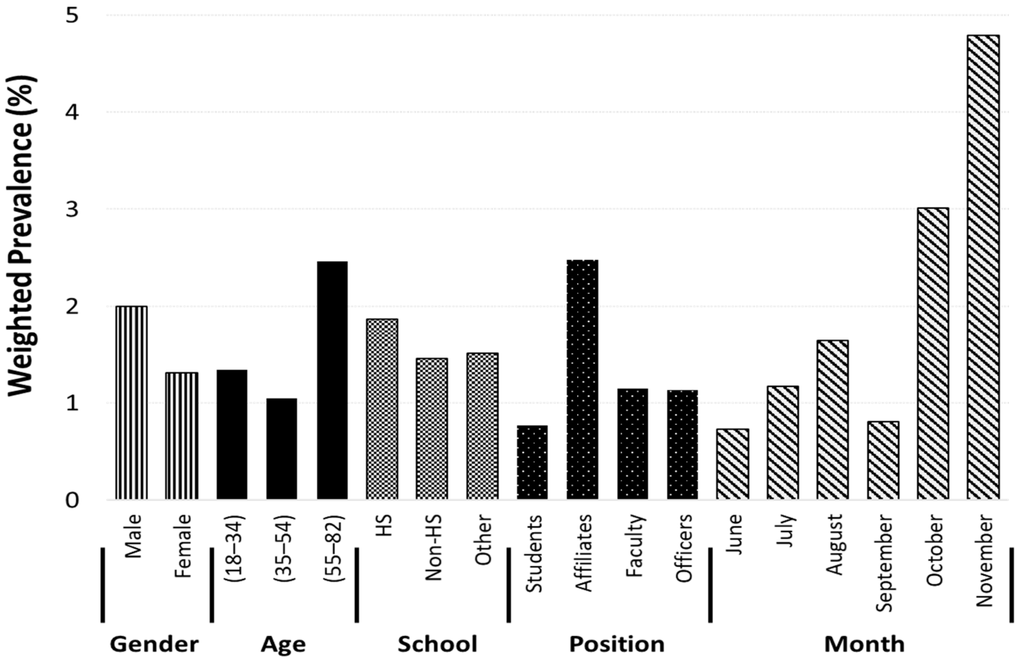

Figure S1. Weighted seroprevalence for age and test performance of anti-SARS CoV-2 N-protein per gender, age group (in years), School of NKUA (HS: Health Sciences, Non-HS: Non-health sciences), position at the NKUA and per month (June-November 2020).
